# Phylogenetic evidence of extensive spatial mixing of diverse HIV-1 group M lineages within Cameroon but not between its neighbours

**DOI:** 10.1093/ve/veae070

**Published:** 2024-09-02

**Authors:** Célestin Godwe, Oumarou H Goni, James E San, Nelson Sonela, Mérimé Tchakoute, Aubin Nanfack, Francioli K Koro, Christelle Butel, Nicole Vidal, Ralf Duerr, Darren P Martin, Tulio de Oliveira, Martine Peeters, Marcus Altfeld, Ahidjo Ayouba, Thumbi Ndung’u, Marcel Tongo

**Affiliations:** Center of Research for Emerging and Re-Emerging Diseases (CREMER), Institute of Medical Research and Study of Medicinal Plants (IMPM), Yaoundé, PO Box. 906 Yaoundé, Cameroon; Department of Biochemistry, University of Douala, Douala, PO Box. 24157 Douala, Cameroon; Center of Research for Emerging and Re-Emerging Diseases (CREMER), Institute of Medical Research and Study of Medicinal Plants (IMPM), Yaoundé, PO Box. 906 Yaoundé, Cameroon; Department of Microbiology, Faculty of Sciences, University of Yaoundé 1, Yaoundé, PO Box. 812 Yaoundé, Cameroon; KwaZulu-Natal Research Innovation and Sequencing Platform, Nelson R. Mandela School of Medicine, University of KwaZulu-Natal, Durban 4001, South Africa; Duke Human Vaccine Institute, Duke University, Durham, NC 27710, United States; Center of Research for Emerging and Re-Emerging Diseases (CREMER), Institute of Medical Research and Study of Medicinal Plants (IMPM), Yaoundé, PO Box. 906 Yaoundé, Cameroon; Chantal BIYA International Reference Centre for Research on HIV/AIDS prevention and management (CIRCB), Yaoundé PO Box. 3077 Yaoundé, Cameroon; Weill Cornell Medical College, Department of Medicine, Cornell University, New York, NY 10021, United States; Programmes de Santé et développement au sein du Groupement de la Filière Bois du Cameroun, PO Box 495, Yaoundé, Cameroon; Chantal BIYA International Reference Centre for Research on HIV/AIDS prevention and management (CIRCB), Yaoundé PO Box. 3077 Yaoundé, Cameroon; Department of Biochemistry, University of Douala, Douala, PO Box. 24157 Douala, Cameroon; TransVIHMI, Université de Montpellier, IRD, INSERM, 911 Avenue Agropolis, Montpellier, Montpellier cedex 34394, France; TransVIHMI, Université de Montpellier, IRD, INSERM, 911 Avenue Agropolis, Montpellier, Montpellier cedex 34394, France; Department of Medicine, Division of Infectious Diseases and Immunology, NYU Grossman School of Medicine, New York, NY 10016, United States; Vaccine Center, NYU Grossman School of Medicine, New York, NY 10016, United States; Department of Microbiology, NYU Grossman School of Medicine, New York, NY 10016, United States; Department of Integrative Biomedical Sciences, Institute of Infectious Disease and Molecular Medicine, University of Cape Town, Observatory, Cape Town 7700, South Africa; KwaZulu-Natal Research Innovation and Sequencing Platform, Nelson R. Mandela School of Medicine, University of KwaZulu-Natal, Durban 4001, South Africa; Centre for Epidemic Response and Innovation (CERI), School of Data Science and Computational Thinking, Stellenbosch University, Stellenbosch 7600, South Africa; TransVIHMI, Université de Montpellier, IRD, INSERM, 911 Avenue Agropolis, Montpellier, Montpellier cedex 34394, France; Universitätsklinikum Hamburg-Eppendorf, Hamburg, Hamburg 20251, Germany; Center of Research for Emerging and Re-Emerging Diseases (CREMER), Institute of Medical Research and Study of Medicinal Plants (IMPM), Yaoundé, PO Box. 906 Yaoundé, Cameroon; TransVIHMI, Université de Montpellier, IRD, INSERM, 911 Avenue Agropolis, Montpellier, Montpellier cedex 34394, France; HIV Pathogenesis Programme, The Doris Duke Medical Research Institute, University of KwaZulu Natal, Durban 4013, South Africa; Africa Health Research Institute (AHRI), Durban 4001, South Africa; Ragon Institute of MGH, MIT and Harvard University, Cambridge MA 02139, United States; Division of Infection and Immunity, University College London, London WC1E 6BT, United Kingdom; Center of Research for Emerging and Re-Emerging Diseases (CREMER), Institute of Medical Research and Study of Medicinal Plants (IMPM), Yaoundé, PO Box. 906 Yaoundé, Cameroon; HIV Pathogenesis Programme, The Doris Duke Medical Research Institute, University of KwaZulu Natal, Durban 4013, South Africa

**Keywords:** HIV-1 group M (HIV-1M), viral diversity, evolution, phylogenetic, Cameroon

## Abstract

From the perspective of developing relevant interventions for treating HIV and controlling its spread, it is particularly important to comprehensively understand the underlying diversity of the virus, especially in countries where the virus has been present and evolving since the cross-species transmission event that triggered the global pandemic. Here, we generate and phylogenetically analyse sequences derived from the *gag-protease* (2010 bp; *n* = 115), partial *integrase* (345 bp; *n* = 36), and *nef* (719 bp; *n* = 321) genes of HIV-1 group M (HIV-1M) isolates sampled between 2000 and 2022 from two cosmopolitan cities and 40 remote villages of Cameroon. While 52.4% of all sequenced viruses belonged to circulating recombinant form (CRF) 02_AG (CRF02_AG), the remainder were highly diverse, collectively representing seven subtypes and sub-subtypes, eight CRFs, and 36 highly divergent lineages that fall outside the established HIV-1M classification. Additionally, in 77 samples for which at least two genes were typed, 31% of the studied viruses apparently had fragments from viruses belonging to different clades. Furthermore, we found that the distribution of HIV-1M populations is similar between different regions of Cameroon. In contrast, HIV-1M demographics in Cameroon differ significantly from those in its neighbouring countries in the Congo Basin (CB). In phylogenetic trees, viral sequences cluster according to the countries where they were sampled, suggesting that while there are minimal geographical or social barriers to viral dissemination throughout Cameroon, there is strongly impeded dispersal of HIV-1M lineages between Cameroon and other locations of the CB. This suggests that the apparent stability of highly diverse Cameroonian HIV-1M populations may be attributable to the extensive mixing of human populations within the country and the concomitant trans-national movements of major lineages with very similar degrees of fitness; coupled with the relatively infrequent inter-national transmission of these lineages from neighbouring countries in the CB.

## Introduction

Cameroon was the likely site of cross-species transmission that yielded HIV-1 group M (HIV-1M) (Gao et al. 1999, Keele et al. 2006). It is known that shortly after transmission to humans ∼1920 (1909–30) (Faria et al. 2014), the progenitor of HIV-1M began to diversify extensively into numerous variants. The absence of geographical barriers likely meant that these ‘pre-subtype’ variants circulated throughout the Congo Basin (CB) region, initiating localized epidemics, some of which may have remained confined within small groups of infected people (Gray et al. 2009, Tongo et al. 2018). The virus arrived in the city of Kinshasa in the Democratic Republic of Congo (DRC) in the 1920s (Korber et al. 2000, Salemi et al. 2001, Faria et al. 2014) where, by the 1950s, repeated human-to-human transmissions and iatrogenic interventions within the city had probably yielded a diverse pool of ‘proto-subtype’ variants, including some lineages that further diversified into what were determined in the 1990s to be distinct HIV-1M subtypes (Faria et al. 2014).

Following this diversification and the global spread of HIV-1M, many variants that had been circulating outside Cameroon were likely reintroduced back into the country either as single founder lineages or as multiple introductions. These reintroduced lineages are potentially circulating within Cameroon together with early diverging indigenous Cameroonian lineages that never left the country in sufficient numbers to have been sampled elsewhere in the world (Tongo et al. 2021). Possibly because of this, Cameroon, like other countries in the CB, has one of the genetically most diverse HIV epidemics in the world. Specifically, in urban areas and rural settlements across the tropical rainforest regions of the country, we and others have found numerous different subtypes, circulating recombinant forms (CRFs), unique recombinant forms (URFs), and other difficult-to-classify genetic variants (Brennan et al. 2008, Carr et al. 2010, Tongo et al. 2013, Banin et al. 2019).

Although CRF02_AG accounts for the majority of virus isolates in all studies describing the HIV-1M diversity in Cameroon, it remains undetermined whether the distribution of other viral lineages varies from region to region in the country. Given the extreme diversity of the Cameroonian HIV-1M epidemic, it would therefore be of great interest to test for regional variations in the prevalence of less common HIV-1M variants and lineages. Of particular interest would be comparisons of lineage prevalence across urban and rural regions of Cameroon spanning the equatorial rainforest regions where it is believed that HIV-1M first began infecting humans. In addition, it is also expected that the true breadth of HIV-1M diversity in Cameroon would only ever be known once viruses are analysed from a number of active infections that substantially exceed one minus the frequency of the least prevalent lineage (Willis 2019).

Improvements in our understanding of the genetic diversity, evolutionary history, and population-level patterns of HIV transmission within HIV diversity hotspots like Cameroon will inform the optimal design and implementation of future global HIV prevention strategies. Towards this end, we use sequences from the gag-protease*,* nef*,* and integrase genes to further understand the diversity of viruses underpinning the HIV-1M epidemic in Cameroon. More specifically, we determine the distribution of HIV-1M lineages and the geographical clustering of these lineages within Cameroon and between Cameroon and its neighbouring countries.

## Materials and methods

### Study participants

**Figure 1.**
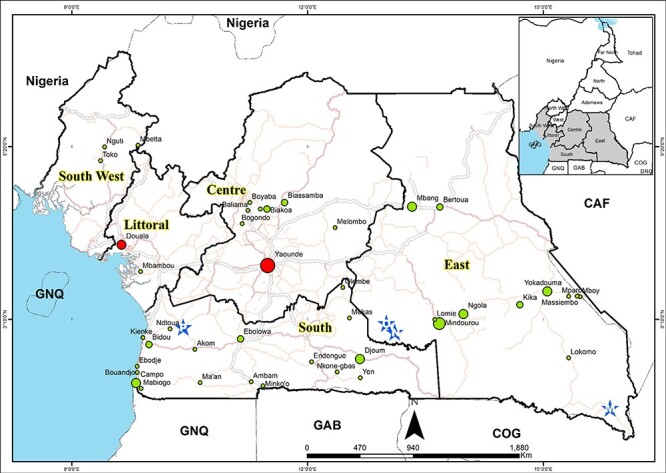
Map of the equatorial rain forest regions of Cameroon illustrating the sites where samples were collected: red circles represent city sampling sites; green circles represent remote villages sampling sites; and blue stars represent the sites where HIV-1 group M (M), group N (N), group O (O), and group P (P) reservoirs were identified.

Plasma samples were obtained from people living with HIV (PLWH) residing in remote or rural locations spanning the CB region of Cameroon, including locations where reservoirs of HIV-1 groups M, N, O, and P were previously discovered (Fig. 1) during health surveys that we carried out in 2000, 2012, 2013, 2021, and 2022. These samples were supplemented with HIV-infected plasma samples collected in 2007, 2008, 2009, 2017, and 2018 from two government blood bank centres located in the two most populous Cameroonian cities: Yaoundé and Douala. The study was approved by the National Ethics Committee of the Cameroonian Ministry of Health (No. 2019/04/1156/CE/CNERSH/SP). All PLWHs were antiretroviral therapy naïve based on a self-reporting questionnaire, but no data on age, gender, or risk factors for HIV were available.

### Geographic mapping of samples

We first entered all the study sites and places where the reservoir of HIV-1 groups M, N, O, and P were identified into an Excel worksheet (Excel worksheet 97-2003). We then imported and converted this spreadsheet as a converted table into a shapefile in the ArcMap interface of the ArcGIS 10.8 package. Finally, we superimposed roads and administrative boundaries of the different regions of interest, obtained from the 2019 National Institute of Cartography, Yaoundé Cameroon database. The hydrographic network and boundaries of African countries were downloaded from OpenStreetMap (OpenStreetMap contributors, 2023)

### Viral RNA extraction and specific gene amplification

RNA was extracted from plasma samples using either Abbott RealTime HIV-1 Extraction Kits (Abbott Molecular Inc., Des Plaines, IL, USA) or QIAamp® Viral RNA Mini Extraction Kits (Qiagen, Hilden, Germany); this was done according to the respective manufacturer protocols.

Complementary DNA (cDNA) was generated using the ImProm-II™ Reverse Transcription System kit (Promega, Madison, WI, ‎USA) according to the manufacturer’s protocol. The *gag-protease, nef*, and *integrase* genome fragments were amplified using non-subtype-specific HIV-1M primers for these genes, producing a 2010-bp fragment spanning the whole *gag-protease* region, a 700-bp fragment spanning the entire *nef* region, and a 396-bp fragment spanning the *integrase* region of the *pol* gene. All PCRs (Polymerase Chain Reaction) were performed using the ROCHE Expand High Fidelity kit (Roche-Mannheim, Germany). First- and second-round reactions were performed using 1 µl of dNTP (10 mM), 5 µl of 10× buffer plus MgCl_2_ (15 mM), 2 µl of each primer (10 µM), 0.75 µl of Expand High Fidelity Enzyme (5 U/µl), water, and template, in a total reaction volume of 50 µl.

For gag-protease PCR, the first-round cycling conditions were as follows: 94°C for 2 min, 10 cycles of 94°C for 15 s, 52°C for 30 s, 72°C for 1 min, followed by 15 cycles of 94°C for 15 s, 55°C for 2 min, 72°C for 1.5 min, and then 72°C for 7 min. The second-round cycling conditions consisted of 94°C for 2 min, followed by 35 cycles of 94°C for 15 s, 55°C for 30 s, 72°C for 1.5 min, and then 72°C for 7 min. The first-round primers were as follows: Gag+1 forward (HXB2: 675–697; 5ʹ-GAGGAGATCTCTCGACGCAGGAC-3ʹ) and 3ʹrvp reverse (HXB2: 2712–2735; 5ʹ-GGCAAATACTGGAGTATTGTATGG-3ʹ). The second-round reaction was with long forward (LgFw) primers (HXB2: 695–789; 5ʹ-GACTCGGCTTGCTGAAGCGCGCACGGCAAGAGGCGAGGGGCGGCGACTGGTGATACGCCAAAAATTTTGACTAGCGGAGGCTAGAAGGAGAGAGATGGG-3ʹ) and long reverse (LgRw) primers (HXB2: 2605–2704; 5ʹ-GGCCCAATTTTTGAAATTTTTCCTTCCTTTTCCATTTCTGTACAAATTTCTACTAATGCTTTTATTTTTTCTTCTGTCAATGGCCATTGTTTAACTTTTG-3ʹ), as previously described by Godwe et al. (2024).

For *nef*, the cycling conditions were the same for both first and second rounds, consisting of 94°C for 2 min followed by 35 cycles of 94°C for 15 s, 55°C for 15 s, 72°C for 50 s and then 72°C 7 min. The nef first round primers were: Nef outer5-le (HXB2: 8513–8533; 5ʹ-GTGCCTCTTCAGCTACCACCG-3ʹ and Nef outer3-3e (HXB2: 9488–9508; Reverse primer 5ʹ-AGCATCTGAGGGTTAGCCACT-3ʹ). The *nef* second round primers were: NEF8746_Sgrl_Ascl_F (HXB2: 8736-8772; 5ʹ- AGAGCACCGGCGCGCCTCCACATACCTASAAGAATMAGACARG-3ʹ) and Nef 9474_Sacll_Clal_R 3-7e (HXB2: 9449-9491; 5ʹ-GCCTCCGCGGATCGATCAGGCCACRCCTCCCTGGAAASKCCC-3ʹ).

For *integrase*, PCR touchdown cycling conditions for the two rounds were also the same and consisted of 94°C for 3 min followed by 10 cycles of 92°C for 20 s, 50°C minus 0.5°per cycle for 30 s, 72°C for 30 s and 35 cycles of 92°C for 20 s, 50°C for 30 s, 72°C for 30 s, and then 72°C for 5 min. The integrase first round primers were PolOR (HXB2: 4958–4978; 5ʹ-ACBACYGCNCCTTCHCCTTTC-3ʹ) and polis4 (HXB2: 4158–4180; 5ʹ-CCAGCNCACAAAGGNATAGGAGG-3ʹ). The second round primers were Uni2 (HXB2: 4781–4806; 5ʹ-CCCCTATTCCTCCCCTTCTTTTAAAA-3ʹ) and polis2 (HXB2: 4410–4436; 5ʹ-TGGCARATRGAYTGYACNCAYNTRGAA-3ʹ) (Courgnaud et al. 2002).

### Sequencing and assembly

Positive PCR products were purified using the QIAquick PCR purification kit (Qiagen, Inc., Toronto, ON, Canada) and then sequenced by Sanger technology for the entire *nef* and *integrase* and partially for the *gag-protease* products using the Applied Biosystems 3500 Genetics Analyzer (Life Technologies, Thermo Fisher Scientific^®^, UK). Another set of *gag-protease* PCR products was sequenced using Illumina MiSeq (Illumina, San Diego, CA, USA) next-generation sequencing (NGS) platforms as described in Godwe et al. (2024). For Sanger sequencing, we used the second round primers for nef and integrase products, while for the gag-protease, a set of seven primers were used and included gf2331 (HXB2: 2331–2350; 5ʹ-GGAGCAGATGATACAGTATT-3ʹ); GF100-1817.18 (HXB2: 1817–1834; 5ʹ-TAGAAGAAATGATGACAG-3ʹ); GR1981 (HXB2: 1960–1981; 5ʹ-CCTTGCCACAGTTGAAACATTT-3ʹ); GR1981 (HXB2: 1960–1981; 5ʹ-CCTTGCCACAGTTGAAACATTT-3ʹ); SQ16RC (HXB2:1078–1098; 5ʹ-CTTGTCTAGGGCTTCCTTGGT-3ʹ); G00 (HXB2: 764–782; 5ʹ-GACTAGCGGAGGCTAGAAG-3ʹ); and GAS4R (HXB2: 1462–1481; 5ʹ-GGTTCTCTCATCTGGCCTGG-3ʹ). Sequences reads were assembled using either ChromasPro version 2.1 (Technelysium Pty Ltd, Australia) or SeqmanPro version 15 Software (DNAstar, Madison, WI, USA). For the *gag-protease* NGS sequencing, libraries were prepared using the DNA Prep kit (Illumina, San Diego, CA, USA) with 100 ng of amplicons each. The libraries were sequenced using the MiSeq v2 reagent kit (500 cycles) on the MiSeq platform (Godwe et al. 2024). The newly generated sequences were submitted to the GenBank database under accession numbers OQ989637–OQ989751 for the *gag-protease* sequences, OQ677029–OQ677064 for the *integrase* sequences, and OQ677065–OQ677083, OQ677085–OQ677088, OQ677090–OQ677137, OQ677140–OQ677182, OQ677184–OQ677201, OQ677203–OQ677219, OQ677221–OQ677222, OQ677224–OQ677267, OQ677269–OQ677275, OQ677277–OQ677343, and OQ677345–OQ677396 for the *nef* sequences.

### Assembly of sequence datasets

The newly generated sequences were grouped with reference sequences, representative of known global HIV-1M genetic diversity; this was achieved by constructing maximum likelihood (ML) trees with all available sequences for each of the genome regions using the GTR+I+G nucleotide substitution model (determined to be the best fit model using online Server IQTree (http://iqtree.cibiv.univie.ac.at) (Nguyen et al. 2015). For subtypes/CRF clades with >15 available sequences, we discard all but one sequence from each of the clades (Tongo et al. 2015). The resulting sequence sets (672 for the nef gene, 412 for the gag-protease gene, and 415 for the integrase gene) comprised (i) sequences from all the classified subtypes, sub-subtypes, and CRFs and (ii) all sequences of presently unclassified HIV-1M lineages (designated as U) that were available in the Los Alamos National Laboratory HIV database (http://hiv-web.lanl.gov/content/hiv-db) on October 2020. A multiple sequence alignment was constructed for each gene using the online implementation of MAFFT version 7 (https://mafft.cbrc.jp/alignment/server/). The alignments were manually refined using MEGA 7 (Kumar et al. 2016) and AliView (Larsson 2014).

### HIV-1M distribution

To infer the phylogenetic relationships between the newly generated sequences, an ML phylogenetic tree was constructed for each of the *gag-protease, nef*, and *pol* genes in two different stages. In the first stage, the different trees were constructed using all the reference subtypes, sub-subtypes, CRFs, unclassified sequences, and newly generated sequences. These phylogenetic trees were subsequently refined by removing reference sequences from the clades that did not contain any of the newly generated sequences; leaving a data set made up of (i) the newly generated sequences and the reference sequences with which these clustered in the initial trees and (ii) all available sequences generated by others for viruses previously sampled in the CB region. The ML phylogenetic trees were inferred using the GTR+I+G nucleotide substitution model (determined to be the best fit model using IQ Tree (Nguyen et al. 2015) online server (http://iqtree.cibiv.univie.ac.at). The robustness of tree branches was assessed through ultrafast bootstrap analysis (1000 resampling iterations) with Shimodaira–Hasegawa approximate likelihood ratios ≥0.99 (Guindon et al. 2005) using the IQTree online server (http://iqtree.cibiv.univie.ac.at). The phylogenetic trees were rooted using two HIV-1 group P sequences. MEGA 7 (Kumar et al. 2016) was used to display the ML trees. Subtype, sub-subtype, or CRF designations of the newly determined sequences were made based on the designations of the previously classified sequences with which they clustered within the ML trees. However, newly determined sequences that could not be assigned to any of the known HIV-1M subtype or CRF clades were classified as divergent sequences and included (i) those residing on isolated branches basal to subtrees containing previously defined HIV-1M subtype or CRF lineages or (ii) those branching near to, but above, the root nodes of subtrees containing previously defined HIV-1M subtype or CRF lineages. We appreciated that these divergent sequences might represent recombinant viruses (which are known to at times) branch basal to the known clades of HIV-1M phylogenetic trees. Therefore, the divergent sequences were referred to as divergent/recombinants (Tongo et al. 2015).

### Analysis of phylogenetic clustering of some HIV-1M lineages within Cameroon and between Cameroon and its neighbours

To examine the clustering of Cameroonian HIV-1M sequences, we constructed Bayesian maximum clade credibility (MCC) trees for each dataset, i.e. for g*ag-protease, nef*, and *integrase* genomic region datasets. We considered three administrative regions (Centre, East, and South) and two cosmopolitan cities (Douala and Yaounde) that we grouped to form a single location; we focused on the dominant CRF02_AG lineages identified within the ML trees that we had constructed earlier. We first inferred new ML trees with all the newly determined CRF02_AG sequences for each gene (*nef*, *gag-prot*, and *pol*) with the same model parameters as the previously constructed trees. We then inspected these ML trees in TempEst version 1.5.3 (Rambaut et al. 2016) to determine whether the sequences contained sufficient temporal signal to warrant the application of a molecular clock model. Sequences that did not conform to the molecular clock were removed from subsequent analysis (Supplementary Fig. S3a–f). To determine the dispersal dynamics of the sampled viruses, we estimated time-calibrated phylogenies using the software package BEAST version 1.10.4 (Suchard et al. 2018) and the GTR +G nucleotide substitution model. To determine the most appropriate coalescent model, we performed model testing by path sampling. Specifically, we set the number of path steps to 50 and the length of the Markov chain Monte Carlo (MCMC) chain for each power posterior to 500 000. We tested two parametric models (strict and constant clock) and one nonparametric model (Skygrid). We compared the marginal likelihoods or Baye’s factors for each model, and the best-performing model was selected (see Supplementary Table S1). A chain length of 100M generations was set for each tree, and parameters were logged every 10 000 steps. MCMC convergence was assessed by calculating effective sample size (ESS) values of the runs. MCMC chains were checked for convergence and proper mixing using Tracer version 1.7.1 (Rambaut et al. 2018). ESS values >200 were obtained for all relevant model parameters. Finally, the MCC trees were summarized from the MCMC samples using TreeAnnotator after discarding 10% or more as determined by Tracer of sampled MCMC states as burn-in. The resulting trees were then visualized using Gggplot2 and Ggtree (Wickham 2016, Yu 2020). The best-performing clock and demographic models were a relaxed clock Skygrid model for the *nef* data set and a relaxed clock exponential growth rate model for both the *gag-prot* and *pol* data sets. Sequences from Cameroon were annotated with colours, indicating their locations of origin (Cities: turquoise blue, Centre: blue, East: light purple, and South: red).

The same procedure was followed to determine the spatial distribution of HIV lineages circulating between Cameroon and its neighbouring countries. For these analyses, we only considered the major circulating lineages for which members of the lineages were sampled in both Cameroon and its neighbours; CRF11_cpx between Cameroon and Central African Republic (CAF), subtype G between Cameroon and COG; and CRF02_AG between Cameroon and both Gabon (GAB) and Equatorial Guinea (GNQ). We subsequently retrieved *protease* gene sequences from countries Cameroon and its neighbours (Protease was the only gene for which sufficient numbers of sequences were available across all these countries) from the Los Alamos National Laboratory HIV-1M sequence database. Since CRF02_AG, CRF11_cpx, and G sequences from Cameroon were overrepresented in the data bank compared to the same lineages in neighbouring countries, including all the Cameroonian sequences in the data set would have biased the analysis in favour of detecting clusters in Cameroon (i.e. the probability of epidemiologically linked samples being included in the analysis would have been higher in Cameroon than in its neighbours), we down-sampled the Cameroonian sequences from these lineages to include only a representative selection of the known diversity of these lineages found in Cameroon (as described earlier): 98 CRF02_AG sequences to match with 90 from GAB and 65 from GNQ; 105 for the CRF11_cpx to match with 99 from the CAF and 23 for subtype G to match with 23 from the COG. Sequence alignment and BEAST analyses were carried out as for the within-Cameroon comparison. Here, the best-performing clock and demographic models were relaxed clock exponential growth for both the *prot_CRF02_AG* and *prot_CRF11_cpx* datasets, and strict clock exponential growth for the *prot_G* dataset. Each sequence was also coloured in the trees according to its country of origin.

### Correlation tests between sequence sampling locations and phylogenetic clustering

To test whether there were discernible degrees of phylogenetic clustering by geographic location, we performed correlation tests between sequence sampling locations and phylogenetic clustering with the Bayesian tip-association significance testing (BaTS) program (Parker et al. 2008). This program tests the null hypothesis that character states are randomly distributed on the tips of the tree by generating a null posterior distribution to compare the observed and expected degrees of clustering by chance. Three test statistics were produced: (i) the parsimony score (PS) (Parker et al. 2008); (ii) the association index (AI) (Wang et al. 2001), for which low scores indicate high degrees of association between a trait (regions of Cameroun) and phylogenetic clustering; and (iii) the monophyletic clade (MC) size, which indicates the proportion of MCs sharing a character trait. A significance level of *P* < .05 for any of the PS, AI, and MC statistics was considered significant evidence for an association between sampling location and phylogenetic clustering. A null distribution of these statistics was determined using the posterior distribution of the MCC trees obtained from the BEAST analysis described earlier. This ensured that the association tests accounted for phylogenetic uncertainty (Parker et al. 2008). For our analyses with BaTS, 10 000 trees from the posterior distribution were used, and 1000 permutations of the test statistics were performed.

## Results

### Demographic and sample collection

We generated and phylogenetically analysed sequences from 395 HIV-1M viruses derived either from the *nef* (719 bp; *n* = 321), *integrase* (345 bp; *n* = 36), or *gag-protease* (2010 bp; *n* = 115) genes; for the latter gene, while 46 viruses were sequenced using the Sanger platform, 69 were generated by NGS technology. These viruses were sampled in the two most cosmopolitan cities of Cameroon, Douala and Yaoundé (*n* = 137 samples), and 40 remote villages from five administrative regions spanning the equatorial rainforest area of Cameroon; this included the Centre region (seven villages and *n* = 21 samples), the Littoral region (one village and *n* = 2 samples), the East region (11 villages and 149 samples), the South region (18 villages and *n* = 79 samples), and the Southwest region (three villages and *n* = 7 samples) (Fig. 1). The number of samples by time and location is summarized in Table 1. Of 395 samples, 77 had two genes typed. There were 71 samples with *gag-protease* and *nef* sequences, three with *gag-protease* and *integrase* sequences, and three with *nef* and *pol* sequences. The studied genes were selected to facilitate ongoing downstream phenotyping experiments aimed at understanding how these genes’ functional/biological characteristics contribute to the uneven distribution of the circulating HIV-1M clades in Cameroon.

**Table 1.** Distribution of samples per year of collection.

Year of sampling	Number of samples (*n*)
2000	58
2007	39
2008	23
2009	44
2012	107
2013	112
2017	1
2018	1
2021	1
2022	9

### Distribution of HIV-1M lineages in Cameroun

#### Global distribution of HIV-1M lineages in Cameroun

**Figure 2.**
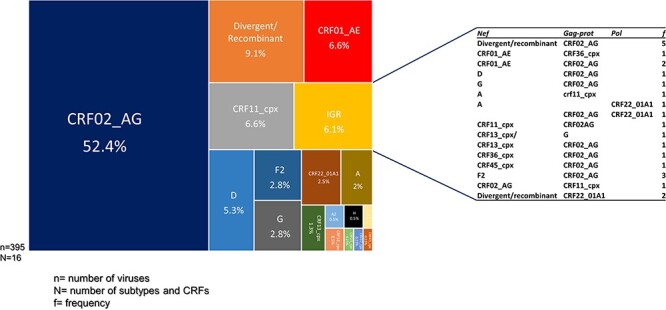
Distribution of HIV-1 group M subtypes and recombinants in Cameroon: the TreeMap represents the 395 viruses from the three studied genes, gag*-prot*, *nef*, and *integrase*. The genetic composition of the IGRs is shown in the table; *N* represents the number of subtypes and CRFs, *n* represents the number of sequenced viruses, and *f* represents the frequency of each IGR.

Phylogenetic analyses of these sequences revealed that they clustered within seven subtypes and sub-subtypes and nine CRF lineages (Supplementary Fig. S1a–c). As expected, the composition of the Cameroonian HIV-1M epidemic is dominated by recombinant viruses with the CRF02_AG lineages accounting for 52.4% of the identified viruses. Other CRFs included -01_AE (6.6%), -11_cpx (6.6%), -13_cpx (1.3%), -18_cpx (0.5%), -22_01A1 (2.5%), -25_cpx (0.25%), -37_cpx (0.25%), and -45_cpx (0.25%). The analysed sequences also clustered with subtypes A (2%), D (5.3%), G (2.8%), and H (0.5%) and sub-subtypes F2 (2.8%), A1 (0.25%), and A2 (0.5%) (Fig. 2; Table 2). Additionally, we observed a high frequency of CRF01_AE, mainly because all CRF22_01A1 sequences are embedded within the CRF01_AE cluster in the *nef* gene.

**Table 2.** Distribution of HIV-1 group M (HIV-1M) lineages in the three studied genes (*nef*, *gag-protease*, and *integrase*) in Cameroon.

HIV-1M lineages	*nef* (%)	g*ag_protease* (%)	*Integrase* (%)	Total (%)
A	10 (3.1)	0 (0)	0 (0)	8 (2)
A1	1 (0.3)	0 (0)	0 (0)	1 (0.25)
A2	1 (0.3)	0 (0)	1 (2.8)	2 (0.5)
D	18 (5.6)	4 (3.5)	1 (2.8)	21 (5.3)
F2	11(3.4)	2 (1.7)	2 (5.6)	11 (2.8)
G	10 (3.1)	6 (5.2)	0 (0)	11 (2.8)
H	2 (0.6)	0 (0)	0 (0)	2 (0.5)
CRF01_AE	32 (10)	2 (1.7)	2 (5.6)	26 (6.6)
CRF02_AG	160 (49.8)	75 (65)	22 (61)	207 (52.4)
CRF11_cpx	22 (6.8)	9 (8)	2 (5.6)	26 (6.6)
CRF13_cpx	6 (1.9)	0 (0)	1 (2.8)	5 (1.3)
CRF18_cpx	2 (0.6)	0 (0)	1 (2.8)	2 (0.5)
CRF22_cpx^a^		11 (9.6)	4 (11)	10 (2.5)
CRF25_cpx	1 (0.3)	0 (0)	0 (0)	1 (0.25)
CRF36_cpx	1 (0.3)	1 (0.9)	0 (0)	0 (0)
CRF37_cpx	0 (0)	1 (0.9)	0 (0)	1 (0.25)
CRF45_cpx	2 (0.6)	0 (0)	0 (0)	1 (0.25)
Divergent/recombinant	42 (13)	4 (3.5)	0 (0)	36 (9.1)
IGR^b^				24 (6.1)
Total	321	115	36	395

a CRF22_01A1 sequences are imbedded within the CRF01_AE subtree in the *nef* gene.

b Intergene recombinant.

Furthermore, for 24/77 of the samples from which two gene sequences were analysed, 24 sequences were classified as belonging to different clades from one another corresponding to 6.1% of the 395 studied viruses. They were identified as intergene recombinants (IGRs) (Fig. 2).

Finally, 36 sequences (9.1%) represent lineages outside the established HIV-1M classification. These unclassified sequences are either recombinants or divergent nonrecombinant lineages (Fig. 2). Among these divergent viruses, we found a sequence (CMA235) that grouped basal to the subtype C subtree in the *gag-protease* and *nef* trees (Supplementary Fig. S1a and b). We subsequently made two trees (as highlighted in the Materials and methods section) rooted to HXB2 and with the representatives of the subtype C diversity and subtype-C fragments from CRF92_C2U and CRF93_cpx, two recently identified CRFs with highly divergent subtype C fragments (Villabona Arenas et al. 2017). The newly generated sequence was still basal to the subtype C subtree in both genes (Supplementary Fig. S2). In addition, two other divergent/recombinant sequences were represented by two genes: while YKL126 branched basal to the subtree containing sequences belonging to CRF19_cpx in *gag-protease* and to CRF36_cpx in *nef*, MBG054 branched basal to subtrees containing sequences belonging to a group of unknown sequences, subtype H and CRFs -04_cpx and -27_cpx in the in *nef* tree and branched basal to all sequences in the *gag-protease* tree (Supplementary Fig. S2).

Analyses of the HIV-1M distribution across the studied genes revealed that the proportion of the major clade, CRF02_AG, decreased with the increased number of sequenced viruses as shown with *nef* and *integrase*: 49.8% of 321 analysed sequences in *nef* vs 61% of 36 sequences in *integrase*. In addition, although g*ag-protease* has more than twice the number of analysed sequences compared to *integrase*, it displays the same pattern of identified lineages (09); moreover, the proportion of CRF02_AG is even greater (65%) in the latter compared to 61% in the former (Table 2).

#### Distribution of HIV-1M in Cameroun at the regional level

**Figure 3.**
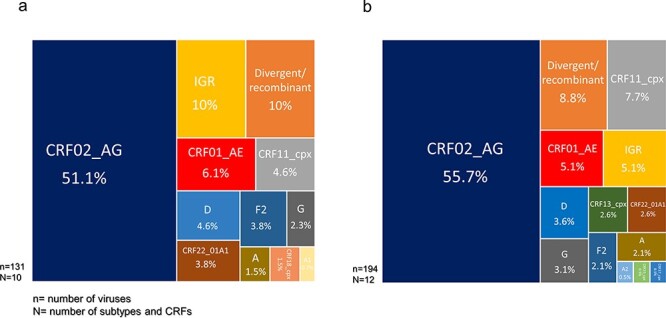
Distributions of HIV-1 group M subtypes and recombinants in Cameroon according to sampling locations: distribution of subtypes and CRFs in (a) cities and within (b) rural areas. *N* represents the number of subtypes and CRFs and *n* represents the number of sequenced viruses.

To study the evolution of virus diversity in the main cosmopolitan cities and rural area communities of the equatorial forest of Cameroon where the virus was likely circulating since before the onset of the global pandemic in ∼1920 (Faria et al. 2014), we compared the demographics of HIV-1M populations in the two settings. For this purpose, we selected viruses that were sampled during approximately the same period; 2007–09 for the cosmopolitan cities (*n* = 131) and 2012/2013 for the remote villages (*n* = 194). We observed that similar main HIV-1M lineages (pure subtypes and CRFs) circulated in the remote areas (12 lineages) and the cosmopolitan cities (10 lineages). Collectively, CRFs -01_AE, -02_AG, -11_cpx, and -22_01A1 and subtypes A, D, G, and F2 accounted for 82% and 77.8% of all the viruses sampled in remote areas and the cities, respectively: with similar frequencies of the different lineages being noted in both settings (Fig. 3, Table 3). In addition, there was no statistically significant difference between the two settings with respect to the numbers of sampled sequences that phylogenetically either branched basal to the known subtype and CRF lineages or branched close to the basal nodes of known subtype or CRF lineages (Table 2). Finally, when two genes were characterized from the same virus, there were twice IGR lineages sampled in the cosmopolitan areas than in the rural areas (10% vs 5.1% of cases), although this difference was not statistically significant (Table 3).

**Table 3.** Distribution of HIV-1 group M (HIV-1M) lineages between remote and cosmopolitan areas in Cameroon.

	Frequency (%)		Statistic	
Subtypes	Remote areas	Cities	*P*-value	Significance
A (*n* = 6)	2.1	1.5	.72	NS
A1(*n* = 1)	0	0.7	.22	NS
A2 (*n* = 1)	0.5	0	.41	NS
D (*n* = 13)	3.6	4.6	.65	NS
F2 (*n* = 9)	2.1	3.8	.34	NS
G (*n* = 9)	3	2.3	.67	NS
CRF01_AE (*n* = 18)	5.1	6.1	.70	NS
CRF02_AG (*n* = 175)	55.4	51.1	.45	NS
CRF11_cpx (*n* = 21)	7.7	4.6	.26	NS
CRF13_cpx (*n* = 5)	2.6	0	.64	NS
CRF18_cpx (*n* = 2)	0	1.5	.83	NS
CRF22_01A1 (*n* = 10)	2.6	3.8	.52	NS
CRF25_cpx (*n* = 1)	0.5	0	.41	NS
CRF37_cpx (*n* = 1)	0.5	0	.41	NS
Divergent/recombinant (*n* = 30)	8.7	10	.71	NS
IGR (23)	5.1	10	.09	NS
Total (325)	100	100		

### Distribution of HIV 1M lineages between Cameroon and its neighbouring countries

**Figure 4.**
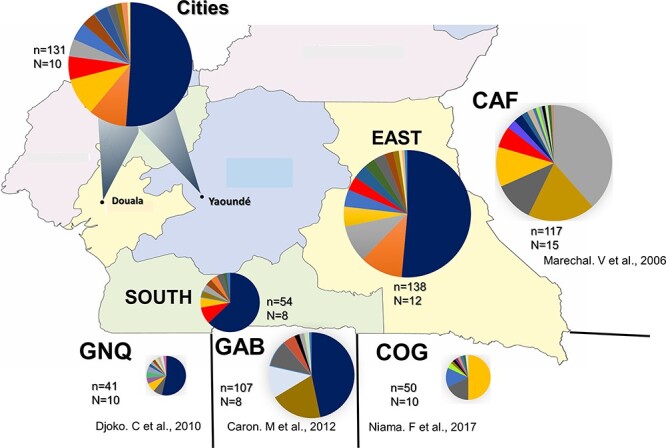
Geographic distribution of subtypes and recombinants among HIV-1 group M subtypes and recombinants sampled in Cameroon and its neighbouring countries (Central African Republic or CAF, Equatorial Guinea or GNQ, Gabon or GAB, and Republic of Congo or COG).

In the absence of substantial differences in the patterns of HIV-1M subtypes/CRFs between remote and urban areas of Cameroon, we compared the HIV-1M distribution in Cameroon with those of neighbouring countries in the CB. We considered the South administrative region of Cameroon, which borders GNQ and GAB, and the East region, which borders the COG and CAF. We used HIV-1M sequence data generated in these Cameroonian regions and from the most extensive studies done in the neighbouring countries (Marechal et al. 2006, Djoko et al. 2010, Caron et al. 2012, Niama et al. 2017). Although CRF02_AG is the major circulating lineage in both the neighbouring GNQ and GAB and the South region of Cameroon, the frequencies of different major lineages in the South region of Cameroon are more similar to those found in other parts of Cameroon than to those found in neighbouring countries (Fig. 4). Similarly, if only considering the Cameroonian sequences sampled in the East region of Cameroon, the frequencies of the different major lineages are more similar to those of other parts of Cameroon than to those found in the neighbouring CAF and COG; the main reason for this is that, in the CAF, the frequency of CRF11_cpx (38%) is far higher than in Cameroon (7%), whereas in COG, a higher frequency (50%) of URFs is found than in Cameroon (31%) (Fig. 4). It is also worth noting that, although all these countries have very diverse HIV-1M epidemics, the frequencies of different lineages are vastly different (Fig. 4).

### Phylogenetic clustering of CRF02_AG viruses within Cameroon

**Figure 5.**
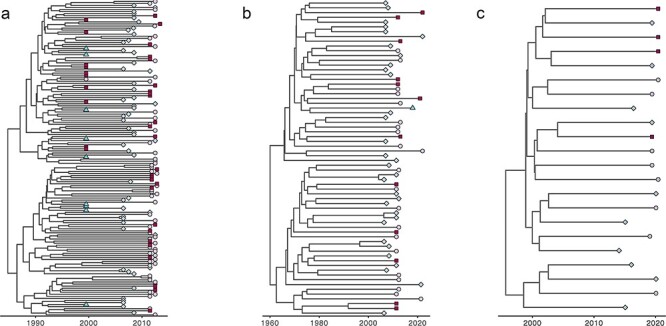
MCC trees of the HIV-1 group M CRF02_AG sequences of different genes of sequences sampled from different regions of Cameroon: the best-performing clock and demographic models were a relaxed clock Skygrid model for the *nef* dataset and a relaxed clock exponential growth rate model for both the *gag-prot* and *pol* datasets. (a) *nef* gene, (b) *gag-prot* gene, and (c) *integrase* gene. The trees are temporally scaled such that distances between points represent 10 years (a and c) and 20 years (b) of evolution for these new Cameroonian sequences. As the legend indicates, each specific region is represented by a unique colour. Turquoise = centre; blue = city; purple = East; and brickred = South. Sequences from different regions are often clustered with sequences from other regions.

We also looked for evidence of barriers to the spread of HIV-1M lineages between the regions of Cameroon. As stated in the Materials and methods section, we focused on the dominant CRF02_AG lineages identified with the initial ML trees (Supplementary Fig. S1a–c). As shown in the CRF02_AG Bayesian MCC *gag-protease, nef*, and *integrase* trees, no sequences from any region formed an MC from deep branches within the trees. Rather, all the sequences generated from viruses sampled in the Centre, East, and South regions and the two cosmopolitan cities (Cities) were spread throughout the different trees (Fig. 5a–c). To detect evidence of more subtle phylogenetic clustering of sequences by sampling locations, we used BaTS. This analysis also failed to reveal any significant associations between the phylogenetic clustering of CRF02_AG sequences and the locations in Cameroon where they were sampled in the three studied genes (Table 4). This lack of geographical structure within the CRF02_AG phylogeny implies the absence of major barriers to dispersing HIV-1M lineages within Cameroon.

**Table 4.** BaTS for spatial structuring of CRF02_AG populations across different locations in Cameroon.

Statistic	Observed mean	Lower 95% CI	Upper 95% CI	Null mean	Lower 95% CI	Upper 95% CI	Significance
*nef*							
AI	9.780	8.701	10.854	12.862	11.588	14.131	0.000
PS	67.245	63.000	71.000	78.328	73.607	82.795	0.000
MC (City)	1.067	1.000	2.000	1.170	1.000	1.919	1.000
MC (East)	3.002	2.000	4.000	2.945	2.124	4.042	0.431
MC (South)	4.350	3.000	5.000	3.177	2.247	4.234	0.094
MC (Centre)	2.166	2.000	3.000	2.306	1.790	3.154	0.831
*gag-protease*							
AI	5.059	4.439	5.692	5.549	4.633	6.394	0.177
PS	29.008	27.000	31.000	31.997	29.299	34.352	0.037
MC (South)	1.000	1.000	1.000	1.011	1.000	1.007	1.000
MC (City)	3.113	3.000	4.000	3.480	2.414	4.937	0.723
MC (East)	2.862	2.000	3.000	2.214	1.508	3.053	0.061
MC (Centre)	2.321	2.000	4.000	1.594	1.011	2.206	0.226
*integrase*							
AI	1.686	1.224	2.138	1.602	1.179	2.010	0.615
PS	9.922	9.000	11.000	9.861	8.411	11.043	0.528
MC (City)	1.646	1.000	2.000	2.237	1.496	3.175	0.644
MC (East)	1.888	1.000	2.000	1.807	1.190	2.847	0.332
MC (South)	1.146	1.000	2.000	1.179	1.000	1.950	1.000

### Spatial distribution of CR02_AG, CRF11_cpx, and subtype G lineages circulating between Cameroon and its neighbouring countries

**Figure 6.**
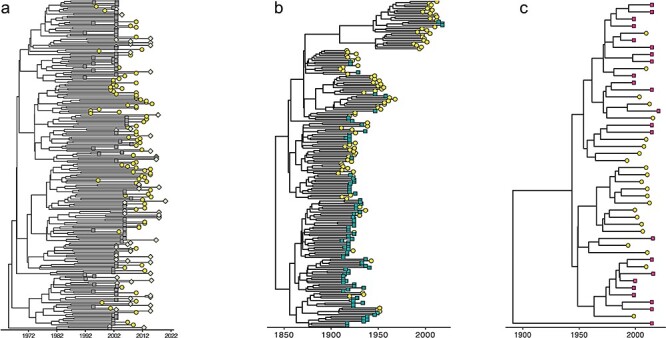
MCC trees inferred from protease sequences depicting the relationship between the epidemic in Cameroon and its neighbours in the CB: the best-performing clock and demographic models were relaxed clock exponential growth for both the *prot_CRF02_AG* and *prot_CRF11_cpx* datasets and strict clock exponential growth for the *prot_G*. (a) HIV-1 group M CRF02_AG sequences sampled from Cameroon, GNQ, and GAB; (b) HIV-1 group M CRF11_cpx from Cameroon and CAF; and (c) HIV-1 group M subtype G from Cameroon and the Congo Republic (COG). The trees are temporally scaled such that distances between points represent 10 years (a) and 50 years (b and c) of evolution for these sequences. Each country is represented by a unique colour and shape. Yellow circle = Cameroon; green diamond = GNQ; grey square = GAB, blue triangle = CAF; and inverted red triangle = Congo Republic.

Overall, the CRF02_AG MCC tree showed clear geographical structure with sequences from Cameroon, GAB, and GNQ forming distinct clusters in the tree (Fig. 6a). Similarly, the subtype G tree indicated that, globally, sequences sampled in the COG and Cameroon grouped in clusters according to their countries of origin (Fig. 6c). The CRF11_cpx tree showed the strongest evidence of geographical clustering. This tree showed two big distinct clusters, each made of sequences originating from Cameroon or CAF (Fig. 6b). The correlation tests between sequence sampling locations and phylogenetic clustering were carried out using BaTS. These analyses indicated significant clustering of sequences according to location, supporting the hypothesis that there is likely only limited movement of viruses in the CRF02_AG, CRF11_cpx, and subtype G lineages across the borders of Cameroon (Table 5).

**Table 5.** BaTS for geographical structuring across different countries within the CB that neighbour Cameroon (COG, CAF, GAB, and GNQ).

Statistic	Observed mean	Lower 95% CI	Upper 95% CI	Null mean	Lower 95% CI	Upper 95% CI	Significance
*protease* of HIV-1M subtype G sequences from Cameroon and COG							
AI	1.715	1.090	2.316	2.525	1.916	3.095	0.018
PS	10.627	9.000	13.000	15.618	13.351	17.537	0.003
MC (CMR)	10.234	10.000	11.000	2.983	2.285	4.064	0.001
MC (COG)	4.792	3.000	8.000	3.021	2.3147	4.145	0.061
*protease* of HIV-1M CRF11_cpx sequences from Cameroon and CAF							
AI	0.042	0.000	0.314	11.719	11.539	11.810	0.000
PS	1.947	1.000	4.000	68.246	66.823	68.841	0.000
MC (CAF)	77.714	32.000	99.000	4.350	4.255	4.543	0.001
MC (CMR)	78.084	32.000	105.000	4.657	4.552	4.850	0.001
*protease* of HIV-1M CRF02_AG sequences from Cameroon, GAB, and GNQ							
AI	7.753	6.069	9.442	18.713	17.325	20.031	0.0
PS	50.136	41.0	59.0	113.471	108.733	117.757	0.0
MC (GAB)	8.693	4.0	16.0	3.647	3.121	4.356	0.001
MC (GNQ)	7.845	4.0	14.0	3.211	2.724	4.047	0.001
MC (CMR)	16.868	7.0	28.0	2.671	2.249	3.275	0.001

## Discussion

In this study, we used sequences from the *gag-protease, nef*, and *integrase* genes to understand further the diversity of viruses underpinning the HIV-1M epidemic in Cameroon. Although we found the most common lineage of viruses circulating in the country is CRF02_AG, the frequency of this lineage may be overestimated because of the limitation in the genome segments that are usually sequenced. Next, we show that the frequencies of diverse HIV-1M subtype and CRF lineages are similar between cities and remote areas of the equatorial rain forest. Finally, we find no evidence of substantial barriers to viral dispersal within Cameroon and that there are clear viral demographic differences between Cameroon and its neighbours in the CB, which strongly suggest spatial barriers to inter-country viral dispersal.

We and others have previously found that viruses sampled from remote areas of Cameroon included many that belonged to highly divergent lineages that were not classifiable within known subtypes and CRFs (Carr et al. 2010, Tongo et al. 2021). As each of the different highly divergent viral lineages must be circulating at very low frequencies (only individual members in each lineage have ever been detected), it is striking that the proportion of total HIV-1M infections involving these lineages remains constant between the remote and cosmopolitan areas. This is perhaps simply attributable to the fact that areas today considered cosmopolitan were relatively isolated villages when the progenitors of the various HIV-1M lineages began to diversify. Alternatively, the absence of detectable geographical barriers to virus dispersal within Cameroon likely meant that viruses belonging to these highly divergent lineages have circulated (and perhaps continue to circulate) throughout the country.

Two previous studies conducted in Cameroon on samples collected between 1995 and 2015 are noteworthy. Whereas the first study focused on 676 samples from the cities of Douala and Yaoundé (involving sampling between 1996 and 2004), the second focused on 555 samples from villages from the South region of Cameroon (involving sampling between 2011 and 2015) (Brennan et al. 2008, Rodgers et al. 2017). Overall, these studies also failed to detect substantial changes in the proportions of different HIV-1M subtypes and CRFs between the two cosmopolitan areas and remote locations in the South region of Cameroon. In addition, these studies also found similar distributions of circulating variants to those reported here. This, together with our results, suggests that the exceptionally high degrees of HIV-1M diversity occurring in Cameroon have remained stable over decades.

The diversity and apparent demographic stability of HIV-1M lineages in Cameroon and the pervasive presence of rare highly divergent lineages are consistent with the hypothesis that, possibly even before the onset of the global pandemic, multiple HIV-1M lineages cocirculated within Cameroon. This suggests that there was likely minimal competition between viruses in the different lineages to find and infect new hosts and that the present-day frequency differences between lineages in Cameroon reflect a composite of slight differences in both the times when lineages first arrived (or emerged) in Cameroon and the evolutionary fitness within the Cameroonian context of these ‘founder’ viruses. However, while it is expected that there will be at least some lineage-specific differences in evolutionary fitness (Kiguoya et al. 2017), it should be stressed that in Cameroon, there is likely minimal competition between different variants for susceptible hosts, given that only approximately 2.7% (varies from 2.9% in urban to 2.4% in rural areas) of the population is presently HIV+ (Centre de Documentation Numérique du Secteur Santé 2020.). Although evolutionary fitness is unlikely to be the primary determinant of differences in the prevalence of the different major Cameroonian HIV-1M lineages, it remains likely that low evolutionary fitness may account for both the failure of very rare and highly divergent Cameroonian lineages to initiate detectable epidemics outside of Cameroon and the failure of rarer CB subtype and CRF lineages failing to initiate epidemics outside of the CB.

If the difference in the relative frequencies of any given pair of Cameroonian HIV-1M lineages is attributable to differences in their fitness, it might be expected, but would not necessarily be so, that their relative frequencies might remain constant in other regions of the CB where they co-occur. However, the composition of HIV-1M population in different parts of the CB supports the opposite view. For example, whereas we found here that CRF02_AG and subtype A/A1 have a frequency of >50% and 2%, respectively, of all the Cameroonian variants characterized, the two lineages, respectively, account for 6% and 43% of HIV-1M infections in the Democratic COG (Rodgers et al. 2017). Therefore, region-to-region differences in the relative frequencies of HIV-1M lineages would likely be attributable to factors other than differences in viral fitness: primary among these being relative durations of circulation in a region and the relative frequencies of variant imports into a region (Rambaut et al. 2001). Irrespective of variations in the transmission capacities of different HIV-1M lineages, the distribution and relative frequencies of these lineages in Cameroon and other parts of the world are probably at least partly (but potentially predominantly) determined by chance. Specifically, in the absence of direct competition between different viral lineages for hosts, the time when a lineage was first imported into a region and began spreading and the frequency of subsequent independent imports of that same lineage (i.e. viral migrations from other regions) will ultimately have a substantial influence on the prevalence of the virus relative to that of other cocirculating lineages within the region. In one sense, the first arriving lineages with the most frequent imports will be more common than viruses that arrive subsequently. In this respect, before the independence of CB countries and the imposition of restrictions on human movements across national borders, there were likely considerably more opportunities for large-scale spatial mixing of human populations: a factor that is likely to have fostered the dispersal of HIV-1M lineages throughout the CB before the 1950s but constrained it after that. This constraint was well illustrated by data published by Mir et al. (2016). They studied the phylodynamics of the African CRF02_AG lineages and found that the CRF02_AG subepidemics in Cameroon and West Africa were generated from a few introductions in the late 1960s and subsequently spread to yield four clusters, three of which are restricted to Cameroon (Mir et al. 2016). Alternatively, we cannot rule out that sampling bias could have influenced some of our findings; more specifically, it is possible that the low sampling density across Cameroon could have impeded the identification of region-specific sequences clusters.

The ongoing identification and enumeration of the relative prevalence of different HIV-1M lineages in the remote equatorial rainforest regions of Cameroon and major Cameroonian cities are crucial for tracking the progression of diverse HIV-1M subepidemics within this region. The present study substantially expands the known diversity of lineages circulating in a region already well established as an HIV-1M diversity hotspot. It also reveals that the multitude of major lineages found in Cameroon is mostly uniformly distributed throughout the country’s remote rural rainforest regions and urban centres.

## Supplementary Material

veae070_Supp
