# Supplementary material for: Phylogenetic evidence of extensive spatial mixing of diverse HIV-1 group M lineages within Cameroon but not between its neighbours
Source: Virus Evol. 2024 Sep 2;10(1):veae070. doi: 10.1093/ve/veae070 (PMC11463025; doi:10.1093/ve/veae070)
Supplement: veae070_Supp [file veae070_supp.zip › suppl_data/Supplementary_Table_1.docx]

**Supplementary Table 1:** Bayes Factors between different demographic models of HIV-1M lineages within Cameroon and between Cameroon and its neighbours.

| **Dataset** |  | **H0** | **H1** | **BF=(H1-H0)** | **Evidence against H0** | | |  | |
| --- | --- | --- | --- | --- | --- | --- | --- | --- | --- |
| Nef_CRF02_AG | Constant (Strict [H0] vs Relaxed [H1]) | -23372.85 | -23361.08 | 11.77 | *Strong* |  | |  | |
|  | Exponential or Exp (Strict [H0] vs Relaxed [H1]) | -23140.57 | -23137.59 | 2.98 | *Weak* |  | |  | |
|  | Skygrid (Strict [H0] vs Relaxed [H1]) | -23185.38 | -23174.97 | 10.41 | *Strong* |  | |  | |
|  | Constant (Relaxed [H0]) vs Exp (Relaxed [H1]) | -23361.08 | -23137.59 | 223.49 | *Very Strong* |  | |  | |
|  | Skygrid (Relaxed [H0]) vs Exp (Relaxed [H1]) | -23174.97 | -23137.59 | 37.38 | *Very Strong* | Skygrid relaxed | |  | |
|  |  |  |  |  |  |  | |  | |
| Prot_CRF02_AG_CMR-GNQ_GAB | Constant (Strict [H0] vs Relaxed [H1]) | -10091.12 | -10096.94 | -5.82 | *Moderate* |  | |  | |
|  | Exp (Strict [H0] vs Relaxed [H1]) | -9857.76 | -9852.86 | 4.9 | *Moderate* |  | |  | |
|  | Skygrid (Strict [H0] vs Relaxed [H1]) | -9858.47 | -9858.67 | -0.2 | *Weak* |  | |  | |
|  | Constant (Strict [H0]) vs Exp (Relaxed [H1]) | -10091.12 | -9852.86 | 238.26 | *Very Strong* |  | |  | |
|  | Skygrid (Strict [H0]) vs Exp (Relaxed [H1]) | -9858.47 | -9852.86 | 5.61 | *Moderate* | Exponential relaxed | |  | |
|  |  |  |  |  |  |  | |  | |
| Prot_G_COG_CMR | Constant (Strict [H0] vs Relaxed [H1]) | -2790.59 | -2795.83 | -5.24 | *Moderate* |  | |  | |
|  | Exp (Strict [H0] vs Relaxed [H1]) | -2739.46 | -2741.48 | -2.02 | *Weak* |  | |  | |
|  | Skygrid (Strict [H0] vs Relaxed [H1]) | -2757.95 | -2751.4 | 6.55 | *Moderate* |  | |  | |
|  | Constant (Strict [H0]) vs Exp (Strict [H1]) | -2790.59 | -2739.46 | 51.13 | *Strong* |  | |  | |
|  | Skygrid (Relaxed [H0]) vs Exp (Strict [H1]) | -2751.4 | -2739.46 | 11.94 | *Strong* | Exponential strict | |  | |
|  |  |  |  |  |  |  | |  | |
| CRF11_Cpx_CMR_CAF | Constant (Strict [H0] vs Relaxed [H1]) | -8876.57 | -8865.01 | 11.56 | *Strong* |  | |  | |
|  | Exp (Strict [H0] vs Relaxed [H1]) | -8648.11 | -8634.73 | 13.38 | *Strong* |  | |  | |
|  | Skygrid (Strict [H0] vs Relaxed [H1]) | -8670.24 | -8677.86 | -7.62 | *Moderate* |  | |  | |
|  | Constant (Relaxed [H0]) vs Exp (Relaxed [H1]) | -8865.01 | -8634.73 | 230.28 | *Very Strong* |  | |  | |
|  | Skygrid (Strict [H0]) vs Exp (Relaxed [H1]) | -8670.24 | -8634.73 | 35.51 | *Very Strong* | Exponential relaxed | |  | |
|  |  |  |  |  |  |  | |  | |
| Gag_prot_CRF02_AG | Constant (Strict [H0] vs Relaxed [H1]) | -23206.44 | -23208.61 | -2.17 | *Weak* |  | |  | |
|  | Exp (Strict [H0] vs Relaxed [H1]) | -23092.96 | -23092.64 | 0.32 | *Weak* |  | |  | |
|  | Skygrid (Strict [H0] vs Relaxed [H1]) | -23196.73 | -23183.08 | 13.65 | *Strong* |  | |  | |
|  | Constant (Strict [H0]) vs Exp (Relaxed [H1]) | -23206.44 | -23092.64 | 113.8 | *Very strong* |  | |  | |
|  | Skygrid (Relaxed [H0]) vs Exp (Relaxed [H1]) | -23183.08 | -23092.64 | 90.44 | *Very strong* | Exponential relaxed | |  | |
|  |  |  |  |  |  |  | |  | |
| Pol_CRF02_AG | Constant (Strict [H0] vs Relaxed [H1]) | -1819.19 | -1827.24 | -8.05 | *Moderate* |  | |  | |
|  | Exp (Strict [H0] vs Relaxed [H1]) | -1805.31 | -1799.49 | 5.82 | *Moderate* |  | |  | |
|  | Skygrid (Strict [H0] vs Relaxed [H1]) | -1800.68 | -1799.58 | 1.1 | *Weak* |  | |  | |
|  | Constant (Strict [H0]) vs Exp (Relaxed [H1]) | -1819.19 | -1799.49 | 19.7 | *Strong* |  | |  | |
|  | Skygrid (Relaxed [H0]) vs Exp (Relaxed [H1]) | -1799.58 | -1799.49 | 0.09 | *Weak* | Exponential Relaxed or  Skygrid Relaxed | |  | |
| H0 = Null model; H1=alternative model; BF=Bayes Factor (difference between the two marginal likelihoods.). Evidence against H0 or H1  was assessed in the following way: BF<0 indicates positive evidence against H1; 1<BF<3 indicates weak evidence against H0; 3< BF<10 indicates  moderate evidence against H0; BF>10 indicates strong evidence against H0. | | | | | | | | |  |
| Although for Pol_CRF02_AG, both the Exponential and Skygrid performed well, we used the Exponential relaxed clock which was slightly better.  CMR=Cameroon; GNQ=Equatorial Guinea; GAB=Gabon; COG=Republic of Congo; CAF=Central Africa Republic | | | | | | |  | |  |
