# Supplementary figures and images for: Phylogenetic evidence of extensive spatial mixing of diverse HIV-1 group M lineages within Cameroon but not between its neighbours
Source: Virus Evol. 2024 Sep 2;10(1):veae070. doi: 10.1093/ve/veae070 (PMC11463025; doi:10.1093/ve/veae070)

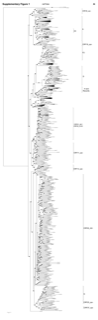

Supplement: veae070_Supp [file veae070_supp.zip › suppl_data/sup.1.png]

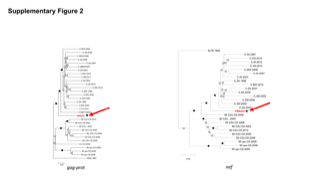

Supplement: veae070_Supp [file veae070_supp.zip › suppl_data/sup.2.png]

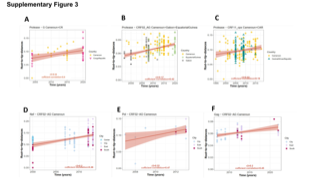

Supplement: veae070_Supp [file veae070_supp.zip › suppl_data/sup.3.png]
